# Optimising sample preparation for FTIR-based microplastic analysis in wastewater and sludge samples: multiple digestions

**DOI:** 10.1007/s00216-021-03331-6

**Published:** 2021-04-23

**Authors:** Serena Cunsolo, John Williams, Michelle Hale, Daniel S. Read, Fay Couceiro

**Affiliations:** 1 School of Civil Engineering and Surveying, Faculty of Technology, University of Portsmouth, Portsmouth, PO1 3AH UK; 2 School of the Environment Geography and Geosciences, Faculty of Science and Health, University of Portsmouth, Portsmouth, PO1 3QL UK; 3 UK Centre for Ecology and Hydrology, Wallingford, OX10 8BB UK

**Keywords:** Wet peroxide oxidation, Organic matter, Microplastics, Wastewater, Recovery, Extraction

## Abstract

The lack of standardised methodologies in microplastic research has been addressed in recent years as it hampers the comparison of results across studies. The quantification of microplastics in the environment is key to the assessment of the potential eco-toxicological impacts that this new category of emerging pollutants could have on terrestrial and aquatic species. Therefore, the need for protocols that are robust, simple and reliable together with their standardisation are of crucial importance. This study has focused on removal of organic matter with Fenton reagent from wastewater and sludge samples. This step of analysis was optimised by implementing a multi-digestion treatment on these samples that have high concentration of complex mixtures of organic matter, which interfere with microplastic enumeration. Moreover, this study targeted the detection of microplastics in the sub-hundred-micron size range due to the potential higher risks associated with smaller-sized particles and the limited data available from previous wastewater research. To show the validity of the method, triplicate samples of raw sewage, final effluent and sludge were independently spiked with two different sizes and types of microplastic polymers. Due to the various analytical stages required for the isolation of microplastics, time is a limiting factor in sample processing. The sequential digestion with Fenton reagent represents an inexpensive and time-efficient procedure for wastewater research providing effective degradation of organic material. These advantages over other currently available methods mean the method is suitable for analysis of large numbers of samples allowing robust monitoring data sets to be generated.

**Supplementary Information:**

The online version contains supplementary material available at 10.1007/s00216-021-03331-6.

## Introduction

Various studies have identified that microplastic (MP) research suffers from the lack of standardised methodologies, from sampling to the characterisation of MP particles in environmental samples [1–7]. The analytical procedures currently used to isolate microplastics (MPs) are dependent on the type of matrix being studied. However, methodologies for extraction and quantification of MPs within the same environmental compartment can vary significantly between studies, hampering the comparison of spatial and temporal patterns of MP abundance. Both sampling strategies and equipment can affect the quality and quantity of MPs reported [2, 8–13]. A wealth of studies on MPs has focused on marine habitats and highlighted the need for future research to investigate further their environmental implications and potential impacts on aquatic organisms. In comparison, the body of knowledge in terrestrial and freshwater ecosystems is limited, although several studies addressed this issue in recent years [6, 14–17]. Among several other land-based sources of MP pollution, wastewater treatment plants (WWTPs) have received attention as they have been identified as terrestrial pathways of MP emissions into aquatic ecosystems [4, 5, 18–27]. Therefore, assessment of the presence and quantification of MPs in effluent discharges and sewage sludge applied to land is a high priority, to enable the risks to be assessed.

It is important that MP detection methodologies are developed that are simple, accurate and efficient, in terms of both time and cost, so that these dynamic and highly variable wastewater system flows can be effectively monitored [28–30]. With regard to the size definition of MPs, no official consensus has yet been reached. However, 1 mm or 5 mm are the most commonly used upper size limits [31–33]. The lower size bound has less consensus, and the definition of nanoplastics has to be taken into account. It has recently been suggested that a cut-off of 1 μm is considered [31, 34, 35]. Some studies have shown that around 60% of MPs found in the final effluent of WWTPs were smaller than 100 μm [4, 21, 23, 25, 36–38]. However, the sub-hundred-micron size range has not been frequently fully investigated because of the large mesh size often used for sample collection as well as technical challenges associated with examining smaller size fractions [6, 12, 29, 39]. The large surface area-to-volume ratio makes the assessment and quantification of smaller-size-range MPs important in understanding their potential eco-toxicological impacts [30, 40–44]. Difficulties in reaching consensus towards a methodological standardisation are hindered by wastewater and sludge being complex and organic-rich matrices. Degradation of organic matter is a major limiting step in the extraction process in terms of the time and costs associated with removal.

 The following section reviews the methods currently applied to decompose the organic component for the detection of MPs in wastewater. It then discusses optimised digestion treatment, followed by density separation with zinc chloride (ZnCl_2_) and analysis of the samples through Fourier transform infrared (FT-IR) spectroscopy. The performance of the chemical characterisation of MPs is imperative to correctly identify and quantify these pollutants in the environment [45–47]. Several techniques can be applied to identify polymer types, with the most widely used being spectroscopy methods such as FT-IR and Raman spectroscopy [47–49]. These techniques are non-destructive and allow for a relatively fast identification of MPs in environmental samples, when used in combination with automated chemical imaging processing to obtain information on abundance, size and polymer type. Moreover, when Raman and FT-IR spectroscopy are coupled with microscopy, particles down to a size of 1 μm and 10–20 μm can be detected, respectively [48–51]. These two analytical tools present their own advantages and disadvantages related to size resolution, measurement time and spectra acquisition modes. They therefore are considered complementary techniques, and the choice mainly resides on the scope of the research [48, 52–54]. In this study, micro FT-IR (μFT-IR) was chosen for MP characterisation as the targeted size range was between 38 and 100 μm.

### Current digestion methods in wastewater research

The quality of an analytical methodology resides in the reliability of each step: from sampling, to organic and inorganic matter removal, and quantitative and qualitative MP identification techniques. Establishing the optimal digestion method for an application requires four factors to be considered: (1) the removal efficiency of the technique and digestion time; (2) the potential damage to MP particles including spectral changes before and after the treatment (if spectroscopic analyses are performed); (3) cost; and finally, (4) the recovery rate in spiking experiments. The most common methodologies in wastewater research are hydrogen peroxide (H_2_O_2_) and Fenton reagent where iron (II) sulphate (FeSO_4_) acts as a catalyst to oxidise the organic component in the presence of H_2_O_2_ [7, 29, 55–58]. This is also referred to as the wet peroxide oxidation (WPO) method that was developed by the National Oceanic and Atmospheric Administration Marine Debris Program [59]. Other widely used methods to extract MPs from biota, sediments and seawater include alkaline (e.g. sodium hydroxide, NaOH; potassium hydroxide, KOH), acidic (e.g. hydrochloric acid, HCl; nitric acid, HNO_3_; perchloric acid, HClO_4_) and enzymatic treatments (e.g. protease-K) [11, 60, 61]. However, it has been shown that acids and alkalis can degrade some plastic polymers such as polyester fibres (PES), nylon (PA), polyethylene (PE), polyvinyl chloride (PVC), polyethylene terephthalate (PET), poly methyl methacrylate (PMMA) and polyurethane (PU) [58, 62–65].

In an important study, Hurley et al. (2018) investigated the impact of four reagents (H_2_O_2_ at 60 °C and 70 °C, Fenton, NaOH and KOH) on organic-rich samples such as soil and sludge matrices. They confirmed that NaOH treatment was inappropriate due to damaging effects on multiple polymer types. KOH caused less damage to MPs (except for polycarbonate), but both KOH and NaOH were found to be unsuitable due to their low efficiency at degrading cellulose and chitin, which are common components of soil and sludge. As for 30% H_2_O_2_, visual changes of the MP polymers were observed at both test temperatures (60 °C and 70 °C), whereas no impact was found when Fenton reagent was used (<40 °C) [55]. Although higher temperatures would promote degradation of organic matter [66], recent work has argued that temperatures above 60 °C cause losses of some plastic polymers. For this reason, a maximum temperature of 50 °C represents a safe cut-off to avoid any losses [22, 64, 67, 68]. Organic degradation rates using the WPO method were also greater than the H_2_O_2_ treatment alone, even at 70 °C. This might be explained by Fenton reagent requiring a low pH (range 2–4) to maximise degradation of organic material, allowing a more successful degradation than H_2_O_2_ treatment alone [55, 68, 69]. This has also been confirmed by visual comparison of different filtered sludge samples after separate treatment with H_2_O_2_ and Fenton reagent [58].

Other studies have also shown that PE, polypropylene (PP), PET and PES, PVC, polystyrene (PS), PU and PA are resistant to WPO, with no change observed to MP size and spectra before and after treatment. Together these polymers account for 92% of global plastic demand [22, 55, 56, 58, 63, 64, 68, 70, 71]. It is important to perform MP recovery experiments to validate the methodology applied, although it is challenging to cover the wide range of polymers present in the environment. Studies using Fenton reagent that have spiked their samples with a known concentration of MPs have obtained relatively high extraction efficiency. Moreover, when WPO is performed in combination with density separation using ZnCl_2_ solution, the combination of both techniques and the order in which they are conducted do not impact recoveries [5, 7, 55, 72–74].

Finally, enzymatic treatments have been proved to be efficient at purifying organic material from wastewater samples. However, these methods have the disadvantage of being costly and time-consuming (taking up to 2 weeks or longer) and are not feasible for the processing of large numbers of samples [7, 38, 39, 75, 76]. Interestingly, this was further investigated by Rodrigues et al. (2018) who compared Fenton reagent alone and in combination with enzymes and concluded that both are equally effective, but WPO represented the ideal compromise due to simplicity and time and cost-effectiveness of the procedure [56].

### Multiple digestions with Fenton reagent

A variation of the WPO technique has been suggested and presented by Dyachenko et al. (2017) [72]. They performed sequential digestions with Fenton reagent and obtained cleaner samples from their experiments, but highlighted the need for validation of this technique with samples spiked with MPs. In wastewater research, spiking experiments have sometimes been conducted with aqueous samples to avoid background interference or by testing manufactured MPs >100 μm [55, 72, 77]. Among those studies where the WPO method was applied and recovery experiments were performed with wastewater and sludge samples, the smallest sizes investigated ranged from 63 to 90 μm (Table 1) [5, 39, 78].

**Table 1:** Reported MP recovery experiments using Fenton reagent. Sample type and spiking materials are shown along with recoveries

Size and polymer type of manufactured MPs used	Type of sample used for recovery	Recovery (%)	Reference
200 μm PS beads	Blank aqueous sample	87	[72]
850–1000 μm PE beads	Sludge and soil	close to 100	[55]
425–500 μm PE beads	Sludge and soil	92–98	[55]
322–395 μm PET fibres	Sludge and soil	79–86	[55]
100 μm PS beads	Raw	77.7	[39]
80–150 μm high-density PE particles	Raw	57.6	[39]
90 μm PS beads	Not specified	89.34	[5]
1000 μm PS beads	Not specified	99.02	[5]
2–4 mm PVC, PP and low-density PE particles	Influent, waste activated sludge and effluent	100	[77]
< 63 μm low-density PE particles	Milli-Q water	93.6	[77]
63–90 μm PA particles	Sludge	52.4	[78]

Another recent study reported using a multiple digestion method, but no details were given about the procedure and recoveries obtained [5]. The aim of this study was to optimise the digestion technique with Fenton reagent, by carrying out multiple digestions based on the work of Dyachenko et al. (2017) [72]. To verify the method’s reliability, three different types of environmental matrices (raw sewage, final effluent and sludge) underwent one or more WPO treatments. Furthermore, recovery experiments with MP sizes down to 38–50 μm were conducted in triplicate on a separate set of samples using the same environmental matrices, which were also subjected to one or multiple digestion cycles. The paper presents a detailed description of the optimised technique that it is simple, cost-effective and more time-efficient compared to current alternative methods.

## Materials and methods

### Microplastic extraction

#### Sample collection and storage

Wastewater and sludge samples were obtained from a municipal secondary wastewater treatment plant in the South of England, UK, that discharges into a nitrogen-sensitive watercourse. It serves a population equivalent of approximately 410,000 and treats almost 190,000 m^3^ d^−1^ of wastewater under dry weather flow. After passing through a 6 mm screen, raw sewage undergoes primary sedimentation followed by secondary treatment in an activated sludge process configured for biological nitrogen removal, with aerobic and anaerobic zones to promote nitrification and subsequent denitrification. The activated sludge mixed liquor is then passed through a final sedimentation tank before the final effluent is discharged. The primary and secondary sludge are mixed before anaerobic digestion, pH amendment and disposal to land. Samples were collected on the same day in August 2019 at three stages of the treatment: raw sewage after 6 mm screens (500 ml), final effluent (2.5 L) and sewage sludge (10 g, wet weight: composed of 5 g of primary sludge and 5 g of secondary sludge).

Compared to other MP studies in wastewater research, smaller sample volumes were taken as it was intended to investigate MPs within the 38–100 μm size range [67, 79]. The sample volumes and weights were informed by preliminary tests (see Supplementary Information (ESM_1), Table S1), which had shown high concentrations of micro-particles. These volumes were appropriate to target the 38–100 μm MP size range and to avoid excessive numbers of MPs being retained on the filters, which would have hampered the identification and characterisation of the particles in the final step of the extraction process (see section "µFT-IR analysis"). On the day of collection, wastewater samples were transported to the laboratory in plastic storage containers (made of high-density PE) and sludge samples in glass bottles, where samples were filtered through 38 μm stainless steel sieves (Endecotts Ltd., London, UK). The material retained on the sieves was rinsed three times with ultrapure water (Milli-Q Direct 8 Water Purification System; Merck Millipore) and stored in the freezer at −18 °C until further analysis. The sludge sample was immediately weighed and stored in the freezer. All samples were stored in plastic jars previously cleaned and rinsed thoroughly three times with ultrapure water. Subsequently, the samples were defrosted at room temperature and poured into glass beakers, which were loosely covered with foil and transferred into an oven set at 50 °C to remove the excess water, until ca. 25–50 ml of sample was left in the beaker.

#### Organic matter removal

The first step of analysis was the removal of the organic matter with Fenton reagent, which was performed under a fume hood. As mentioned above, samples were not completely dried but kept damp as this facilitated the digestion reaction. Raw sewage, final effluent and sludge samples were all treated in the same way. All reagents were freshly prepared each time and filtered (except for H_2_O_2_) prior to analysis to reduce contamination; 100 ml of 0.05 M FeSO_4_ solution (iron II sulphate heptahydrate, ACS reagent, >99%; Sigma-Aldrich) [75] was prepared and poured into the glass beaker, followed by the addition of 100 ml of 30% H_2_O_2_ (hydrogen peroxide 30% *w*/*v*, 100 volumes, Extra Pure SLR, Fisher Chemical; Fisher Scientific). FeSO_4_ was pre-filtered using cellulose nitrate membrane filters (Sartorious™ cellulose nitrate membrane filters, 47 mm, and 0.45 μm pore size). Temperature, pH and H_2_O_2_/FeSO_4_ ratio are important factors that play a key role in the catalytic oxidation. As this combination generates an exothermic reaction, temperature was kept below 50 °C using an ice bath to preserve the MP polymers. A 1 M sodium hydroxide solution (sodium hydroxide, Extra Pure, SLR, pellets, Fisher Chemical; Fisher Scientific), pre-filtered using a PTFE membrane filter (0.2 μm pore size), was used to maintain pH between 3 and 4, to prevent the reduction of soluble iron species reacting with H_2_O_2_ [39, 69]. To avoid overflow of the high volumes of H_2_O_2_ and FeSO_4_, 600 ml glass beakers were used to treat raw and sludge sewage samples, while 400 ml glass beakers were used for final effluent samples. During the digestion, samples were loosely covered with foil. The total reaction time, measured by the presence of visible bubbles in the samples, ranged from half an hour up to 2 h, after which only small bubbles were present. The samples were then left to cool overnight covered with foil. In order to dissolve the excess of ferric precipitates present in the mixture, ca. 10 ml of sulphuric acid was slowly added with a glass pipette to each sample (ca. 250 ml). The solution was gently stirred with the same pipette for a few seconds prior to filtration through a 38 μm sieve to rinse off the reagents. The materials retained on the mesh of the sieve were transferred into a beaker after being rinsed three times with ultrapure water. Hexane treatment, as suggested by Dyachenko et al. (2017), was not used as it could have affected polystyrene and polycarbonate particles [72, 80]. The final effluent sample underwent one digestion cycle, sludge underwent two cycles and raw sample underwent three cycles, due to the visible organic matter present in solution after implementing the first digestion. In each cycle, 100 ml of FeSO_4_ and 100 ml of H_2_O_2_ were added once again to the beakers, and the procedure was repeated.

#### Density separation and filtration

Density separation was performed using zinc chloride solution (ZnCl_2_; 98 + %, extra pure, ACROS Organics™) with a density of 1.7 g cm^−3^ to remove inorganic debris and allow extraction of the heavier polymers [74]. ZnCl_2_ solution was freshly prepared each time and filtered before use over 0.7 μm glass microfiber filters (Fisherbrand™ Microglass Fiber Filter Discs, 47 mm; Fisher Scientific). At the end of the last digestion cycle and after filtration through a 38 μm sieve, samples were rinsed three times with ZnCl_2_ instead of ultrapure water, to prevent a change in density. Samples were first poured into small beakers and then into 100 ml glass separation funnels previously rinsed three times with ZnCl_2_, kept closed with lids and left to settle for a minimum of 15 h, after which 2/3 of the solution was drained out through the valve [81]. The remaining solution was poured into a sieve stack with a 100 μm sieve on top and a 38 μm sieve below to discard the fraction larger than 100 μm. The separation funnels were rinsed three times with ultrapure water to ensure all particles were transferred from the funnel to the sieves. Finally, the samples collected on the 38 μm sieve were rinsed with copious amounts of ultrapure water to wash the ZnCl_2_ off and poured into small glass beakers. However, sometimes, residues of ZnCl_2_ were still present in solution. To avoid any interference with the spectral acquisition, two drops of HCl acid were added with a glass pipette to the sample to dissolve the residual salts. Immediately after, the samples were vacuum-filtered using a 13 mm glass filter holder (Cole-Palmer Advantec 311100 All-Glass Microanalysis filter holder, 13 mm; item #WZ-06644-84) fitted with a 25 mm silver membrane filter (Sterlitech, 5 μm pore size) for the subsequent μFT-IR analysis. After filtration, the silver filters were dried overnight (>15 h) in an oven at 50 °C and then stored in small petri dishes in the dark.

#### μFT-IR analysis

**Fig. 1:**
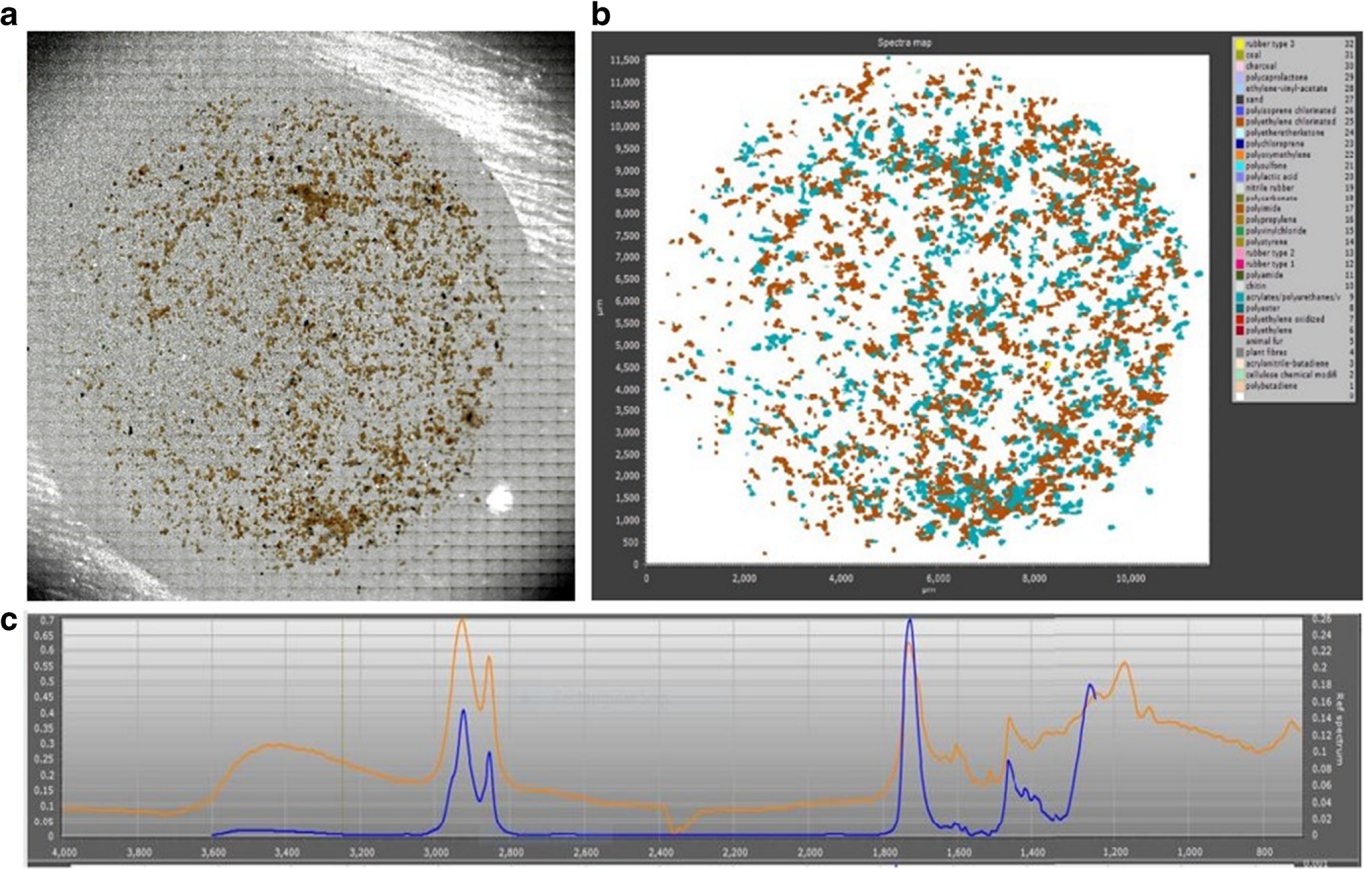
**a** Optical image of the raw sample created by the μFT-IR imaging system; **b** spectra map of the MPs identified in the raw sample; **c** example of a particle spectrum identified as belonging to the acrylates-polyurethanes-varnish polymer group (orange line) and its reference spectrum (blue line)

Once microplastics had been extracted through digestion and density separation, the subsequent analysis steps were performed at the UK Centre for Ecology and Hydrology (UKCEH, Wallingford) and following the protocol reported by Horton et al. (2021) [78]. The spectroscopic analysis was carried out using a PerkinElmer Spotlight™ 400 μFT-IR imaging system, in reflectance mode using a liquid nitrogen-cooled linear array detector, covering the IR spectral range from 4000 to 700 cm^−1^. The spectral resolution was 8 cm^−1^, using four scans per pixel, and the pixel resolution was 25 μm, representing a good compromise between mapping time and signal-to-noise quality of the spectra. This study used 38 μm as lower size cut-off to give good detection at the 25 μm resolution. A background spectrum collection was also carried out on a clean area of the silver filter, and the settings used were the same as for the samples, except for the number of scans per pixel (90 scans/pixel). An optical image of an area of ca. 13 × 13 mm was first generated (Fig. 1a). Because of the size limit of the spectrum image file created by the μFT-IR imaging system, a filter surface area of 11.6 mm × 11.6 mm was mapped, which was equal to 92% of the whole filter area being scanned [82–84]. This was considered sufficient to reliably estimate MP concentrations and polymer types present in the samples [85]. The analysis of one sample on the μFT-IR took between 2.5 and 3 h. Once the spectra map images were acquired and the spectra of the particles generated, they were analysed through siMPle software to obtain information about MP numbers, polymer type and size [84]. An image with a map of the pixels representing the MPs that have been identified was also obtained (Fig. 1b), and the spectrum for each of the MPs was visualised and compared to a spectral reference database (Fig. 1c).

siMPle has been developed by Aalborg University (Denmark) in collaboration with Alfred Wegener Institute (Germany) by combining the software MPhunter [82] with the automated analysis of Primpke et al. 2017 [86]. siMPle is freely available for download together with a reference database (https://simple-plastics.eu/) [84]. In this study, the AAU pipeline has been used for image analysis, and it is based on a score threshold system [78, 81, 82, 84, 87]. This software is used to compare the infrared spectra collected from the μFT-IR against the spectra of the reference database for automated analysis. A score for each particle is obtained based on the quality of this match, ranging from 0.01 (misassignment) to 1 (certain assignment). This score is generated by an algorithm, through which the raw spectra, their first derivatives and their second derivatives are correlated by a Pearson correlation. This yields three Pearson’s correlation coefficients, to which the user assigns global weights (k_0_, k_1_, k_2_) [84]. The score is calculated by using the equation reported in Liu et al. (2019) [82]. In this study, the Pearson’s correlation coefficient for the first probability threshold was set at 0.60, and the default settings of siMPle were used (k_0_ = 0, k_1_ = 1, k_2_ = 1). The second and third probability thresholds, which help define the size of the particle, were left unmodified. At present, there are no guidelines in the literature as to the most appropriate thresholds [81]; therefore, we manually evaluated and compared the spectra across different polymers types by assigning different weights and score thresholds. A threshold setup of 0.60 using the first and the second derivatives of the raw spectra data yielded the best fit.

### Method validation

#### Recovery experiments

In order to validate the multiple digestion steps, recovery experiments (positive controls) were carried out with two polymer types of manufactured MPs of different sizes. As the targeted MP size in this study is in the 38–100 μm range, two sizes of 38–50 μm and 100 μm were chosen for testing. PMMA particles of 38–50 μm in size (Merck Sigma-Aldrich, product no. 463183) and 100 μm PS beads (Merck Sigma-Aldrich, product no. 56969) were separately added to two sets of raw sewage (500 ml), final effluent (2.5 L) and sludge (5 g, wet weight) samples, processed in triplicate. The PS stock solution was prepared by dissolving 100 μl of PS in 100 ml of ultrapure water. For the PMMA stock solution, 0.01 g of PMMA was dissolved in ultrapure water and filtered through the 38 μm sieve, as the presence of PMMA particles smaller than 50 μm (down to 15 μm) was observed under the microscope in preliminary experiments. Particles were collected from the sieve and dissolved in 100 ml of ultrapure water. A 1:50 dilution was made to obtain the final PMMA working solution. The first set of raw, final effluent and sludge samples was spiked with 2 ml of PMMA final working solution, while the second set of samples was spiked with 1 ml of PS stock solution. The stock and final working solutions were stirred gently on a magnetic mixer prior to use. At the same time, separate controls for PMMA and PS particles were performed to limit the margin of variability, as a high variation in the numbers of MPs added to each triplicate sample had been observed in preliminary tests, due to agglomeration and fragmentation. Controls were carried out by vacuum-filtering 2 ml of final working solution and 1 ml of stock solution respectively over black polycarbonate filters (0.2 μm pore size, 25 mm, Whatman™, Nuclepore™) using a 25 mm Millipore glass filter holder to facilitate counting at the microscope.

**Fig. 2:**
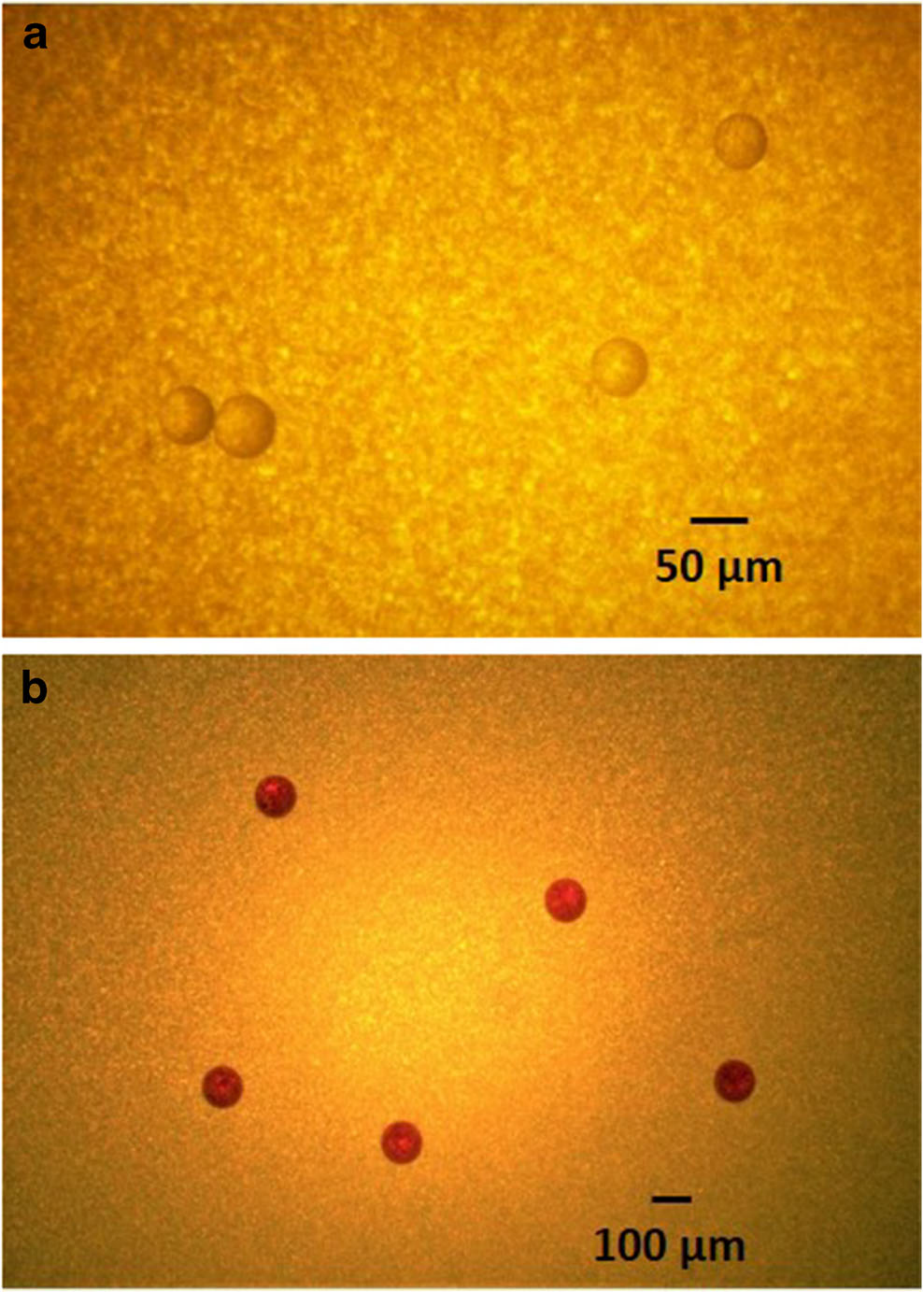
Photos of manufactured MPs used in the recovery experiments taken under the light microscope (Olympus BH2-RFCA): **a** PMMA, ×100 magnification; **b** PS, ×32 magnification

Subsequently, raw, final effluent and sludge samples underwent the digestion and the density separation treatments described in sections "organic matter removal" and "density separation and filtration", followed by the recovery filtration step described above. The filtration through the 100 μm sieve was not performed after density separation to avoid the retention of the 100 μm beads on the sieve. Final effluent samples received one digestion cycle, sludge underwent two cycles and raw samples underwent three cycles, as per section"organic matter removal". Prior to filtration, 40 μl of sodium dodecyl sulphate (SDS; dodecyl sulphate sodium salt, 99%, Acros Organics™) solution (4 g/L) was added to the final extract solution (50 ml) to prevent agglomeration of virgin MP particles. Finally, for both controls and samples, the whole surface of each filter was screened, and particles were visually counted under the light microscope (Olympus BH2-RFCA; ×32 and ×100 magnification for the PS beads and PMMA particles, respectively) (Fig. 2).

#### Procedural blanks and contamination

Careful attention was paid to prevent airborne contamination in the field and the laboratory. Plastic containers and glass bottles for wastewater and sludge collection were covered with lids at all times on site, except for when collection was carried out. Field blanks were taken in triplicate in order to monitor the potential extent of contamination on site by using ultrapure water in glass bottles. In the lab, all surfaces were wiped down with a high-level disinfectant (Chemgene HLD_4_L) prior to the experiments. All treatments were run under a fume hood, except for the initial filtration step through the sieves before sample storage. Lab coats made of 100% cotton were worn at all times. To mitigate airborne contamination, samples, glassware and equipment were always covered with aluminium foil both in and out of the fume hood, except for when the reagents were poured into the beakers. However, we acknowledge that although the use of materials made of plastic was minimised, it could not be completely avoided. All glassware and equipment (e.g. stainless steel sieves and filter rigs) were cleaned using a 2% Decon™ detergent solution (Decon 90; Fisher Scientific) before and after use to prevent cross-contamination among samples. Glassware and equipment were then rinsed thoroughly with reverse osmosis water and then rinsed three times with ultrapure water prior to use. Procedural blanks (in triplicate) were taken by using ultrapure water and underwent two digestion cycles and the same treatment to which the samples were subjected, covering all the steps from sample preparation to filtration in order to assess contamination from plastic containers and air deposition. The blanks were vacuum-filtered using a 13 mm glass filter holder unit (Cole-Palmer Advantec 311100 All-Glass Microanalysis filter holder, 13 mm; item #WZ-06644-84) over 25 mm silver membrane filters (Sterlitech, 5 μm pore size). Both field and procedural blanks were analysed via μFT-IR, and the acquired infrared spectra were run through siMPle software using the same settings reported in section "µFT-IR analysis".

## Results and discussion

### Method validation

**Fig. 3:**
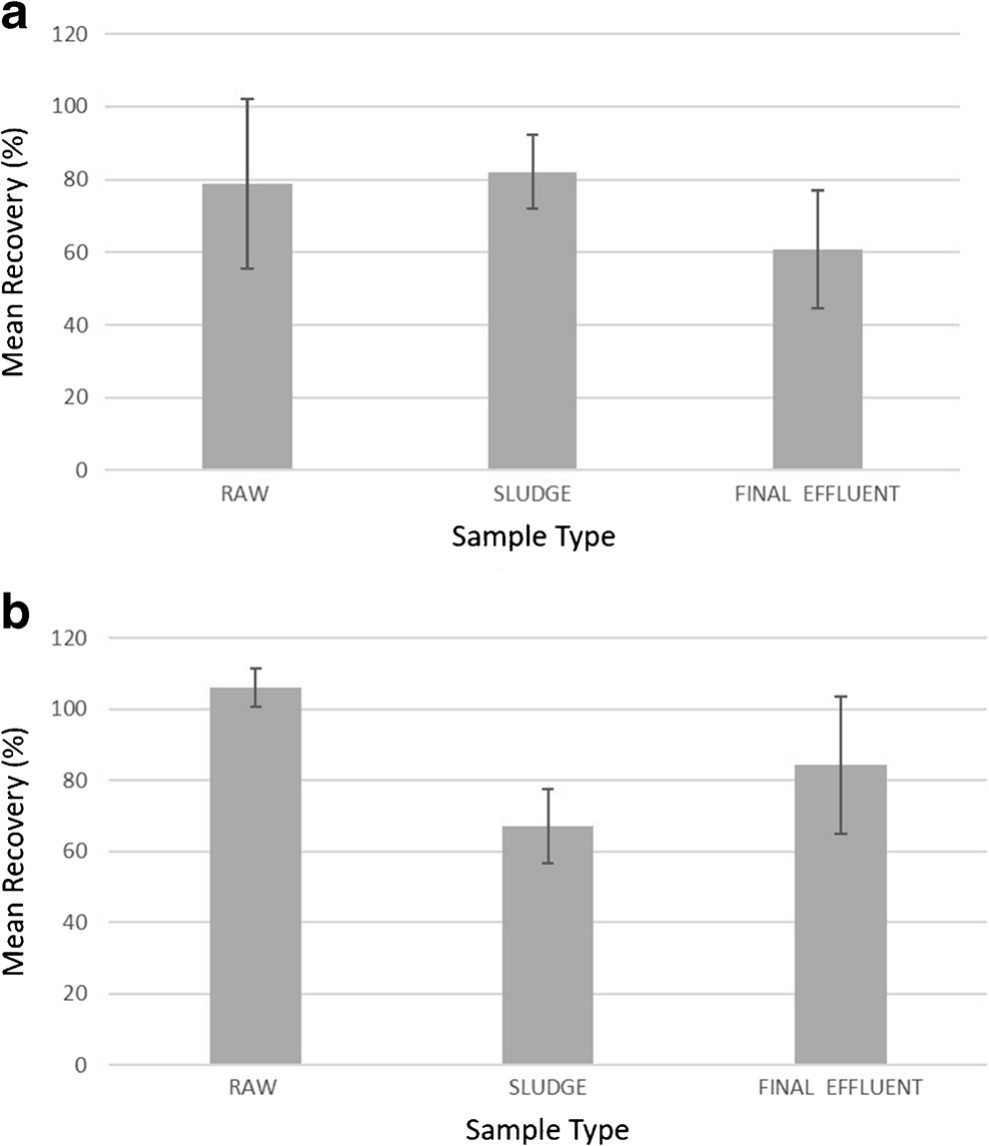
Mean recovery by sample type for **a** PMMA particles and **b** PS beads

Recovery experiments were carried out to assess the efficiency of a multi-digestion-steps procedure with Fenton reagent. Raw, final effluent and sludge samples were separately spiked with PS beads (100 μm) and PMMA particles (38–50 μm), and experiments were performed in triplicates per sample type. A mean count of the MPs added in the controls was calculated within each set of triplicates. This value was then used to obtain the recovery (%) for each replicate based on the count of MPs recovered in the correspondent spiked sample. The mean recovery by sample type for PMMA and PS beads are shown in Fig. 3. In particular, with regard to PMMA particles, the mean recovery for raw, final effluent and sludge was 78.8 ± 23.2%, 60.9 ± 16.3% and 82.2 ± 9.9% (see ESM_1, Table S2), respectively. As for the PS beads, it was 106.1 ± 5.5% for raw wastewater, 84.3 ± 19.4% for final effluent and 67.1 ± 10.3% for sludge (see ESM_1, Table S3). As the spiked concentration was unknown, a control was carried out for each sample to estimate the number of manufactured MPs that were being added. In particular, the recovery for the PS beads in the raw samples was over 100%. This is possibly an artefact of the inherent variability of the spiking process when adding the manufactured MPs. Table S3 (see ESM_1) shows that the number of MPs recovered in the raw samples slightly exceeded the average number counted in the controls. The results of the spiking experiments show that multiple digestions do not cause loss of MPs. The majority of studies that have conducted recovery experiments have tested manufactured particles that were larger than the size range of the MPs being investigated. In this experiment, the MP sizes selected included the lower and upper limit cut-off of the targeted size range (38–100 μm) and underwent all the steps of the analysis.

In order to reduce the inherent variability of preparing controls during the spiking process, a mean value of MPs counted in the controls was used. The recoveries obtained fall within the 60–100% range of recovery efficiency that has been reported in the literature (Table 1). In our study, smaller-sized manufactured particles (38–50 μm) were tested in real environment samples. It is important to note that when performing recovery experiments, the sizes of the spiked MPs should be chosen based on the targeted size range investigated, and that environmental samples should be used instead of clean aqueous solutions (Table 1). The recovery experiments have shown the validity of this method with efficient recovery of the added manufactured MPs. The size of the manufactured particles used in the spiking experiments could affect their recovery, as smaller particles are more likely to adhere to the vacuum filtration unit funnel due to a greater surface charge resulting from their higher surface-to-volume ratio. However, the surface characteristics of MPs present for a long time in the environment might differ from those of virgin plastic. Furthermore, the surface particle properties can also vary based on the polymer type [88]. Therefore, future work is required to explore the influence of polymer type and size on the recovery of manufactured MPs, as well as other factors such as other surfactant solutions and their interactions with both pristine and environmentally weathered MPs. Previous research has used SDS solution, and this has often been coupled with sonication. This may facilitate the recovery of particles [38, 39], but the brittle nature of MPs means that they could potentially break down in an ultrasonic bath. Therefore, the use of more invasive techniques has been avoided in this study [1, 10].

### Analysis of MPs

Here we report the results on the MP concentrations of the non-spiked samples after analysis with siMPle software. Three types of environmental samples were collected and treated with single digestion (final effluent) or multiple digestions (raw and sewage sludge). As mentioned in section "µFT-IR analysis", 92% of the surface area of the filter was analysed via μFT-IR. The total concentration of MPs present in each sample was estimated by extrapolating the number of MPs found to the whole surface area of the filter. Estimates of MP counts ranged from 2102.16 MPs l^−1^ in raw wastewater, to 129.13 MPs l^−1^ in final effluent and 1979.74 MPs g^−1^ of dry weight in sewage sludge (Table 2). Procedural blanks were performed in triplicate, and the mean of MP concentration was 7.25 MPs l^−1^ (Table 2). Field blanks were also analysed to monitor the contamination, and the results show an average of 2.37 MPs l^−1^ (Table 2).

**Table 2:** Estimates of MP counts (per litre or grams of dry weight) obtained in the raw sewage, final effluent and sludge samples examined and average of MPs l^−1^ present in procedural and field blanks

Sample type	Estimate of MPs l^−1^(or MPs g^−1^ of dry weight*)	Average of MPs l^−1^ inprocedural blanks	Average of MPs l^−1^ infield blanks
Raw	2102.16	7.25	2.37
Final effluent	129.13		
Sludge	*1979.74		

The raw data of both samples and procedural blanks obtained from siMPle software on polymer type, shortest and longest dimensions of the particles and their estimated mass and volume are reported in the Supplementary Information (ESM_2). MP concentrations have not been corrected for recovery as only two polymer classes (PS and PMMA) were tested [39]. It is acknowledged that these results do not reflect the behaviour of all the different classes of polymers. Sequential digestions with Fenton reagent could also be applied for batch processing and monitoring purposes. For instance, up to 10 samples (with similar volumes to those used in this study) could be processed by one person in 1 day. If three digestions are performed, it would take a total of 3 days to digest 10 samples. Compared to recent studies where enzyme purification has been performed, the digestion time alone ranged from 4 days up to 13 days depending on the amount and type of enzymes used [38, 39, 76]. In the method presented in this paper, the density separation and filtration of 10 samples over silver filters could be performed in 2 days and the μFT-IR analysis in 2–3 days. Therefore, the total duration of the analysis process for 10 samples would be 7–8 days. Other chemical analysis methodologies for polymer characterisation could be performed as an alternative to μFT-IR or to complement this technique, such as μRaman, pyrolysis-gas chromatography-mass spectrometry and thermos-extraction and desorption gas chromatography-mass spectrometry [89]. However, in the present study, the suitability of silver filters has not been tested with other techniques, as this was beyond the scope of this work. We acknowledge that the processing time for this last step of analysis could vary depending on what method is applied and the research question, subsequently affecting the whole duration of the analysis. For instance, μRaman, compared to μFT-IR, is more time-consuming (up to several days for one sample). This is mainly due to its lower detection thresholds (down to 1 μm), leading to a smaller area of the filter being scanned, and to Raman imaging systems and particle-finding algorithms that are in early stages of development [89]. With regard to thermal degradation methods, they have the disadvantage of not measuring particle size, but the advantage of providing information about chemicals and additives present on MPs. According to Primpke et al. 2020, these methods are less time-efficient compared to Raman and FT-IR [89]. It should be further highlighted that all these methods for the analytical characterisation of MPs are complementary as they differ in the benefits that they offer. Given the scope of this study, μFT-IR was chosen because it provides a reliable and fast polymer characterisation for the analysis of MPs >10 μm, and unlike thermal degradation methods, it is non-destructive.

## Conclusions

This study has demonstrated the optimisation of the digestion treatment with Fenton reagent by performing multiple digestion cycles to target MPs in the sub-hundred-micron size range in wastewater and sludge samples. In order to validate the method, recovery experiments were conducted in triplicate testing two polymer types of different sizes. It has been shown that the WPO treatment performed in multiple cycles represents a valid alternative to current methods and a good compromise as a low-cost and time-efficient procedure, which also preserves the microplastic particles. The development of methodologies that are reliable, simple and relatively fast to perform is important for the accurate detection and quantification of MPs in the environment. Future research could investigate other polymer types and the recovery of smaller manufactured MPs.

## Supplementary Information

ESM 1 (PDF 28 kb)

ESM 2 (XLSX 155 kb)

## Data Availability

All data generated or analysed during this study are included in this published article and its supplementary information files.

## References

[1] Renner G, Schmidt TC, Schram J (2018). Analytical methodologies for monitoring micro(nano)plastics: which are fit for purpose?. Curr Opin Environ Sci Health.

[2] Costa MF, Pinto da Costa J, Duarte AC (2018). Sampling of micro(nano)plastics in environmental compartments: how to define standard procedures?. Curr Opin Environ Sci Health.

[3] Silva AB, Bastos AS, Justino CIL, da Costa JP, Duarte AC, Rocha-Santos TAP (2018). Microplastics in the environment: challenges in analytical chemistry - A review. Anal Chim Acta.

[4] Gatidou G, Arvaniti OS, Stasinakis AS (2019). Review on the occurrence and fate of microplastics in sewage treatment plants. J Hazard Mater.

[5] Long Z, Pan Z, Wang W, Ren J, Yu X, Lin L (2019). Microplastic abundance, characteristics, and removal in wastewater treatment plants in a coastal city of China. Water Res.

[6] Okoffo ED, O’Brien S, O’Brien JW, Tscharke BJ, Thomas KV (2019). Wastewater treatment plants as a source of plastics in the environment: a review of occurrence, methods for identification, quantification and fate. Environ Sci Water Res Technol.

[7] Lusher AL, Munno K, Hermabessiere L, Carr S (2020). Isolation and extraction of microplastics from environmental samples: an evaluation of practical approaches and recommendations for further harmonization. Appl Spectrosc.

[8] Hanvey JS, Lewis PJ, Lavers JL, Crosbie ND, Pozo K, Clarke BO (2017). A review of analytical techniques for quantifying microplastics in sediments. Anal Methods.

[9] Hidalgo-Ruz V, Gutow L, Thompson RC, Thiel M (2012). Microplastics in the marine environment: A review of the methods used for identification and quantification. Environ Sci Technol.

[10] Löder MGJ, Gerdts G, Bergmann M, Gutow L, Klages M (2015). Methodology Used for the Detection and Identification of Microplastics—A Critical Appraisal. Marine Anthropogenic Litter [Internet].

[11] Miller ME, Kroon FJ, Motti CA (2017). Recovering microplastics from marine samples: A review of current practices. Mar Pollut Bull.

[12] Kang P, Ji B, Zhao Y, Wei T (2020). How can we trace microplastics in wastewater treatment plants: A review of the current knowledge on their analysis approaches. Sci Total Environ.

[13] Bretas Alvim C, Mendoza-Roca JA, Bes-Piá A (2020). Wastewater treatment plant as microplastics release source – quantification and identification techniques. J Environ Manag.

[14] Blettler MCM, Abrial E, Khan FR, Sivri N, Espinola LA (2018). Freshwater plastic pollution: recognizing research biases and identifying knowledge gaps. Water Res.

[15] Bucci K, Tulio M, Rochman CM (2020). What is known and unknown about the effects of plastic pollution: A meta-analysis and systematic review. Ecol Appl.

[16] Horton AA, Walton A, Spurgeon DJ, Lahive E, Svendsen C (2017). Microplastics in freshwater and terrestrial environments: evaluating the current understanding to identify the knowledge gaps and future research priorities. Sci Total Environ.

[17] Law KL (2017). Plastics in the marine environment. Annu Rev Mar Sci.

[18] Eriksen M, Mason S, Wilson S, Box C, Zellers A, Edwards W (2013). Microplastic pollution in the surface waters of the Laurentian Great Lakes. Mar Pollut Bull.

[19] Jambeck JR, Geyer R, Wilcox C, Siegler TR, Perryman M, Andrady A (2015). Plastic waste inputs from land into the ocean. Science.

[20] Murphy F, Ewins C, Carbonnier F, Quinn B (2016). Wastewater treatment works (WwTW) as a source of microplastics in the aquatic environment. Environ Sci Technol.

[21] Edo C, González-Pleiter M, Leganés F, Fernández-Piñas F, Rosal R (2020). Fate of microplastics in wastewater treatment plants and their environmental dispersion with effluent and sludge. Environ Pollut.

[22] Bretas Alvim C, Bes-Piá MA, Mendoza-Roca JA (2020). Separation and identification of microplastics from primary and secondary effluents and activated sludge from wastewater treatment plants. Chem Eng J.

[23] Conley K, Clum A, Deepe J, Lane H, Beckingham B (2019). Wastewater treatment plants as a source of microplastics to an urban estuary: removal efficiencies and loading per capita over one year. Water Res X.

[24] Hidayaturrahman H, Lee T-G (2019). A study on characteristics of microplastic in wastewater of South Korea: identification, quantification, and fate of microplastics during treatment process. Mar Pollut Bull.

[25] Kazour M, Terki S, Rabhi K, Jemaa S, Khalaf G, Amara R (2019). Sources of microplastics pollution in the marine environment: importance of wastewater treatment plant and coastal landfill. Mar Pollut Bull.

[26] Ben-David EA, Habibi M, Haddad E, Hasanin M, Angel DL, Booth AM (2021). Microplastic distributions in a domestic wastewater treatment plant: removal efficiency, seasonal variation and influence of sampling technique. Sci Total Environ.

[27] Blair RM, Waldron S, Gauchotte-Lindsay C (2019). Average daily flow of microplastics through a tertiary wastewater treatment plant over a ten-month period. Water Res.

[28] Eerkes-Medrano D, Thompson RC, Aldridge DC (2015). Microplastics in freshwater systems: a review of the emerging threats, identification of knowledge gaps and prioritisation of research needs. Water Res.

[29] Sun J, Dai X, Wang Q, van Loosdrecht MCM, Ni B-J (2019). Microplastics in wastewater treatment plants: detection, occurrence and removal. Water Res.

[30] Hung C, Klasios N, Zhu X, Sedlak M, Sutton R, Rochman CM (2021). Methods matter: methods for sampling microplastic and other anthropogenic particles and their implications for monitoring and ecological risk assessment. Integr Environ Assess Manag.

[31] Frias JPGL, Nash R (2019). Microplastics: finding a consensus on the definition. Mar Pollut Bull.

[32] Bergmann M, Gutow L, Klages M. Marine Anthropogenic Litter: Springer; 2015.

[33] Arthur, C., J. Baker, H. Bamford. Proceedings of the International Research Workshop on the Occurrence, Effects and Fate of Microplastic Marine Debris. Sept 9-11, 2008. [Internet]. NOAA Technical Memorandum NOS-OR&R-30.; 2009. Available from: https://www.repository.library.noaa.gov/view/noaa/2509. Accessed 3 Oct 2020

[34] Gigault J, ter Halle A, Baudrimont M, Pascal P-Y, Gauffre F, Phi T-L (2018). Current opinion: what is a nanoplastic?. Environ Pollut.

[35] Hartmann NB, Hüffer T, Thompson RC, Hassellöv M, Verschoor A, Daugaard AE (2019). Are we speaking the same language? Recommendations for a definition and categorization framework for plastic debris. Environ Sci Technol.

[36] Liu X, Yuan W, Di M, Li Z, Wang J (2019). Transfer and fate of microplastics during the conventional activated sludge process in one wastewater treatment plant of China. Chem Eng J.

[37] Wolff S, Kerpen J, Prediger J, Barkmann L, Müller L (2019). Determination of the microplastics emission in the effluent of a municipal waste water treatment plant using Raman microspectroscopy. Water Res X.

[38] Mintenig SM, Int-Veen I, Löder MGJ, Primpke S, Gerdts G (2017). Identification of microplastic in effluents of waste water treatment plants using focal plane array-based micro-Fourier-transform infrared imaging. Water Res.

[39] Simon M, van Alst N, Vollertsen J (2018). Quantification of microplastic mass and removal rates at wastewater treatment plants applying focal plane array (FPA)-based Fourier transform infrared (FT-IR) imaging. Water Res.

[40] Huvet A, Paul-Pont I, Fabioux C, Lambert C, Suquet M, Thomas Y (2016). Reply to Lenz et al.: quantifying the smallest microplastics is the challenge for a comprehensive view of their environmental impacts. Proc Natl Acad Sci.

[41] Koelmans AA, Bakir A, Burton GA, Janssen CR (2016). Microplastic as a vector for chemicals in the aquatic environment: critical review and model-supported reinterpretation of empirical studies. Environ Sci Technol.

[42] Rochman CM, Lewison RL, Eriksen M, Allen H, Cook A-M, Teh SJ (2014). Polybrominated diphenyl ethers (PBDEs) in fish tissue may be an indicator of plastic contamination in marine habitats. Sci Total Environ.

[43] Strungaru S-A, Jijie R, Nicoara M, Plavan G, Faggio C (2019). Micro-(nano) plastics in freshwater ecosystems: abundance, toxicological impact and quantification methodology. TrAC Trends Anal Chem.

[44] Besseling E, Redondo-Hasselerharm P, Foekema EM, Koelmans AA (2019). Quantifying ecological risks of aquatic micro- and nanoplastic. Crit Rev Environ Sci Technol.

[45] Ivar do Sul JA (2021). Why it is important to analyze the chemical composition of microplastics in environmental samples. Mar Pollut Bull.

[46] Wang W, Wang J (2018). Investigation of microplastics in aquatic environments: an overview of the methods used, from field sampling to laboratory analysis. TrAC Trends Anal Chem.

[47] Renner G, Nellessen A, Schwiers A, Wenzel M, Schmidt TC, Schram J. Data preprocessing & evaluation used in the microplastics identification process: A critical review & practical guide. TrAC Trends Anal Chem. 20191;111:229–38.

[48] Xu J-L, Thomas KV, Luo Z, Gowen AA (2019). FTIR and Raman imaging for microplastics analysis: state of the art, challenges and prospects. TrAC Trends Anal Chem.

[49] Cabernard L, Roscher L, Lorenz C, Gerdts G, Primpke S (2018). Comparison of Raman and Fourier transform infrared spectroscopy for the quantification of microplastics in the aquatic environment. Environ Sci Technol.

[50] Veerasingam S, Ranjani M, Venkatachalapathy R, Bagaev A, Mukhanov V, Litvinyuk D (2020). Contributions of Fourier transform infrared spectroscopy in microplastic pollution research: a review. Crit Rev Environ Sci Technol.

[51] Fortin S, Song B, Burbage C (2019). Quantifying and identifying microplastics in the effluent of advanced wastewater treatment systems using Raman microspectroscopy. Mar Pollut Bull.

[52] Käppler A, Fischer D, Oberbeckmann S, Schernewski G, Labrenz M, Eichhorn K-J (2016). Analysis of environmental microplastics by vibrational microspectroscopy: FTIR, Raman or both?. Anal Bioanal Chem.

[53] Vinay Kumar BN, Löschel LA, Imhof HK, Löder MGJ, Laforsch C (2021). Analysis of microplastics of a broad size range in commercially important mussels by combining FTIR and Raman spectroscopy approaches. Environ Pollut.

[54] Sobhani Z, Zhang X, Gibson C, Naidu R, Megharaj M, Fang C (2020). Identification and visualisation of microplastics/nanoplastics by Raman imaging (i): down to 100 nm. Water Res.

[55] Hurley RR, Lusher AL, Olsen M, Nizzetto L (2018). Validation of a method for extracting microplastics from complex, organic-rich. Environ Matrices Environ Sci Technol.

[56] Rodrigues MO, Gonçalves AMM, Gonçalves FJM, Nogueira H, Marques JC, Abrantes N (2018). Effectiveness of a methodology of microplastics isolation for environmental monitoring in freshwater systems. Ecol Indic.

[57] Tagg AS, Sapp M, Harrison JP, Sinclair CJ, Bradley E, Ju-Nam Y, et al. Microplastic Monitoring at Different Stages in a Wastewater Treatment Plant Using Reflectance Micro-FTIR Imaging. Front Environ Sci. 2020;8. Available from: https://www.frontiersin.org/articles/10.3389/fenvs.2020.00145/full. Accessed 16 Feb 2021

[58] Al-Azzawi MSM, Kefer S, Weißer J, Reichel J, Schwaller C, Glas K (2020). Validation of sample preparation methods for microplastic analysis in wastewater matrices—reproducibility and standardization. Water.

[59] Masura, Baker, Foster & Arthur. Laboratory methods for the analysis of microplastics in the marine environment: recommendations for quantifying synthetic particles in waters and sediments. NOOA Technical Memorandum NOS-OR&R-48 [Internet]. 2015. Available from: https://www.repository.library.noaa.gov/view/noaa/10296. Accessed 6 Sept 2018

[60] Wang Z, Taylor SE, Sharma P, Flury M (2018). Poor extraction efficiencies of polystyrene nano- and microplastics from biosolids and soil. PLoS One.

[61] Zarfl C (2019). Promising techniques and open challenges for microplastic identification and quantification in environmental matrices. Anal Bioanal Chem.

[62] Enders K, Lenz R, Beer S, Stedmon CA (2017). Extraction of microplastic from biota: recommended acidic digestion destroys common plastic polymers. ICES J Mar Sci.

[63] Herrera A, Garrido-Amador P, Martínez I, Samper MD, López-Martínez J, Gómez M (2018). Novel methodology to isolate microplastics from vegetal-rich samples. Mar Pollut Bull.

[64] Munno K, Helm PA, Jackson DA, Rochman C, Sims A (2018). Impacts of temperature and selected chemical digestion methods on microplastic particles. Environ Toxicol Chem.

[65] Li X, Chen L, Ji Y, Li M, Dong B, Qian G (2020). Effects of chemical pretreatments on microplastic extraction in sewage sludge and their physicochemical characteristics. Water Res.

[66] Sujathan S, Kniggendorf A-K, Kumar A, Roth B, Rosenwinkel K-H, Nogueira R (2017). Heat and bleach: a cost-efficient method for extracting microplastics from return activated sludge. Arch Environ Contam Toxicol.

[67] Koelmans AA, Mohamed Nor NH, Hermsen E, Kooi M, Mintenig SM, De France J (2019). Microplastics in freshwaters and drinking water: critical review and assessment of data quality. Water Res.

[68] Treilles R, Cayla A, Gaspéri J, Strich B, Ausset P, Tassin B (2020). Impacts of organic matter digestion protocols on synthetic, artificial and natural raw fibers. Sci Total Environ.

[69] Zhang H, Choi HJ, Huang C-P (2005). Optimization of Fenton process for the treatment of landfill leachate. J Hazard Mater.

[70] Geyer R, Jambeck JR, Law KL (2017). Production, use, and fate of all plastics ever made. Sci Adv.

[71] Tagg AS, Harrison JP, Yk J-N, Sapp M, Bradley EL, Sinclair CJ (2017). Fenton’s reagent for the rapid and efficient isolation of microplastics from wastewater. Chem Commun.

[72] Dyachenko A, Mitchell J, Arsem N (2017). Extraction and identification of microplastic particles from secondary wastewater treatment plant (WWTP) effluent. Anal Methods.

[73] Estahbanati S, Fahrenfeld NL (2016). Influence of wastewater treatment plant discharges on microplastic concentrations in surface water. Chemosphere..

[74] Lo H-S, Xu X, Wong C-Y, Cheung S-G (2018). Comparisons of microplastic pollution between mudflats and sandy beaches in Hong Kong. Environ Pollut.

[75] Lares M, Ncibi MC, Sillanpää M, Sillanpää M (2018). Occurrence, identification and removal of microplastic particles and fibers in conventional activated sludge process and advanced MBR technology. Water Res.

[76] Löder MGJ, Imhof HK, Ladehoff M, Löschel LA, Lorenz C, Mintenig S (2017). Enzymatic purification of microplastics in environmental samples. Environ Sci Technol.

[77] Raju S, Carbery M, Kuttykattil A, Senthirajah K, Lundmark A, Rogers Z (2020). Improved methodology to determine the fate and transport of microplastics in a secondary wastewater treatment plant. Water Res.

[78] Horton AA, Cross RK, Read DS, Jürgens MD, Ball HL, Svendsen C (2020). Semi-automated analysis of microplastics in complex wastewater samples. Environ Pollut.

[79] Covernton GA, Pearce CM, Gurney-Smith HJ, Chastain SG, Ross PS, Dower JF (2019). Size and shape matter: a preliminary analysis of microplastic sampling technique in seawater studies with implications for ecological risk assessment. Sci Total Environ.

[80] Gies EA, LeNoble JL, Noël M, Etemadifar A, Bishay F, Hall ER (2018). Retention of microplastics in a major secondary wastewater treatment plant in Vancouver. Can Mar Pollut Bull.

[81] Johnson AC, Ball H, Cross R, Horton AA, Jürgens MD, Read DS, et al. Identification and quantification of microplastics in potable water and their sources within water treatment works in England and Wales. Environ Sci Technol. 2020. 10.1021/acs.est.0c03211. 10.1021/acs.est.0c03211 32852201

[82] Liu F, Olesen KB, Borregaard AR, Vollertsen J (2019). Microplastics in urban and highway stormwater retention ponds. Sci Total Environ.

[83] Primpke S, Wirth M, Lorenz C, Gerdts G (2018). Reference database design for the automated analysis of microplastic samples based on Fourier transform infrared (FTIR) spectroscopy. Anal Bioanal Chem.

[84] Primpke S, Cross RK, Mintenig SM, Simon M, Vianello A, Gerdts G (2020). EXPRESS: toward the systematic identification of microplastics in the environment: evaluation of a new independent software tool (siMPle) for spectroscopic analysis. Appl Spectrosc.

[85] Mintenig SM, Kooi M, Erich MW, Primpke S, Redondo-Hasselerharm PE, Dekker SC (2020). A systems approach to understand microplastic occurrence and variability in Dutch riverine surface waters. Water Res.

[86] Primpke S, Lorenz C, Rascher-Friesenhausen R, Gerdts G (2017). An automated approach for microplastics analysis using focal plane array (FPA) FTIR microscopy and image analysis. Anal Methods.

[87] Vianello A, Jensen RL, Liu L, Vollertsen J (2019). Simulating human exposure to indoor airborne microplastics using a breathing thermal manikin. Sci Rep.

[88] Filella M (2015). Questions of size and numbers in environmental research on microplastics: methodological and conceptual aspects. Environ Chem.

[89] Primpke S, Christiansen SH, Christiansen SH, Christiansen SH, Cowger W, Frond HD (2020). Critical assessment of analytical methods for the harmonized and cost-efficient analysis of microplastics. Appl Spectrosc.

