# Supplementary material for: Optimising sample preparation for FTIR-based microplastic analysis in wastewater and sludge samples: multiple digestions
Source: Anal Bioanal Chem. 2021 Apr 23;413(14):3789–99. doi: 10.1007/s00216-021-03331-6 (PMC8141486; doi:10.1007/s00216-021-03331-6)
Supplement: Supplementary file 1 — (PDF 28 kb) [file 216_2021_3331_MOESM1_ESM.pdf]

Optimising sample preparation for FTIR-based microplastic analysis in wastewater and sludge samples: multiple digestions

Serena Cunsolo[1], John Williams[1], Michelle Hale[2], Daniel S. Read[3] and Fay Couceiro[1]

[1]School of Civil Engineering and Surveying, Faculty of Technology, University of Portsmouth, Portsmouth, PO1 3AH, U.K.

[2]School of the Environment Geography and Geosciences, Faculty of Science and Health, University of Portsmouth, Portsmouth, PO1 3QL, U.K.

[3]UK Centre for Ecology and Hydrology, Wallingford, OX10 8BB, U.K.

Corresponding author: Serena Cunsolo (serena.cunsolo@port.ac.uk)

Supplementary Information

Table S1 Sample volumes, sample weights and filtration devices used in preliminary tests and final experiments by sample type

	Preliminary Experiments		***Final Experiments***	
**Sample type**	**Sample volume (L) or *weight (g of wet weight)**	**Filtration device used**	**Sample volume (L) or *weight (g of wet weight)**	**Filtration device used**
RAW	1	25 mm glass filter holder unit	0.5	13 mm glass filter holder unit
FIN. EFF.	10	25 mm glass filter holder unit	2.5	13 mm glass filter holder unit
SLUDGE	*30	25 mm glass filter holder unit	*10	13 mm glass filter holder unit

Table S2 Recovery results for PMMA particles added to each triplicate by sample type (raw sewage, final effluent and sludge)

Sample Type	MPs recovered	MPs counted in controls	Average of MPs counted in controls	Recovery (%)	Average of Recovery (%)	SD
RAW 1	48	55		105.11		
RAW 2	32	42	45.67	70.07	78.83	23.17
RAW 3	28	40		61.31		
SLUDGE 1	40	42		76.43		
SLUDGE 2	40	66	52.33	76.43	82.16	9.93
SLUDGE 3	49	49		93.63		
FIN. EFF. 1	40	51		74.53		
FIN. EFF. 2	35	67	53.67	65.22	60.87	16.28
FIN. EFF. 3	23	43		42.86		

Table S3 Recovery results for PS beads added to each triplicate by sample type (raw sewage, final effluent and sludge)

Sample Type	MPs recovered	MPs counted in controls	Average of MPs counted in controls	Recovery (%)	Average of Recovery (%)	SD
RAW 1	76	75		100.00		
RAW 2	82	65	76	107.89	106.14	5.48
RAW 3	84	88		110.53		
SLUDGE 1	19	36		78.08		
SLUDGE 2	16	22	24.33	65.75	67.12	10.34
SLUDGE 3	14	15		57.53		
FIN. EFF. 1	38	44		64.04		
FIN. EFF. 2	61	83	59.33	102.81	84.27	19.44
FIN. EFF. 3	51	51		85.96		
